# Early trajectory of clinical global impression as a transdiagnostic predictor of psychiatric hospitalisation: a retrospective cohort study

**DOI:** 10.1016/S2215-0366(23)00066-4

**Published:** 2023-05

**Authors:** Maxime Taquet, Kira Griffiths, Emily O C Palmer, Sheryl Ker, Christian Liman, Soon Nan Wee, Scott H Kollins, Rashmi Patel

**Affiliations:** a Department of Psychiatry, University of Oxford, Oxford, UK; b Oxford Health NHS Foundation Trust, Oxford, UK; c Holmusk Technologies, New York, NY, USA; d Duke University School of Medicine, Durham, NC, USA; e Akili, Boston, MA, USA; f Department of Psychological Medicine, Institute of Psychiatry, Psychology, and Neuroscience, King's College London, London, UK

## Abstract

**Background:**

Identifying patients most at risk of psychiatric hospitalisation is crucial to improving service provision and patient outcomes. Existing predictors focus on specific clinical scenarios and are not validated with real-world data, limiting their translational potential. This study aimed to determine whether early trajectories of Clinical Global Impression Severity are predictors of 6 month risk of hospitalisation.

**Methods:**

This retrospective cohort study used data from the NeuroBlu database, an electronic health records network from 25 US mental health-care providers. Patients with an ICD-9 or ICD-10 code of major depressive disorder, bipolar disorder, generalised anxiety disorder, post-traumatic stress disorder, schizophrenia or schizoaffective disorder, ADHD, or personality disorder were included. Using this cohort, we assessed whether clinical severity and instability (operationalised using Clinical Global Impression Severity measurements) during a 2-month period were predictors of psychiatric hospitalisation within the next 6 months.

**Findings:**

36 914 patients were included (mean age 29·7 years [SD 17·5]; 21 156 [57·3%] female, 15 748 [42·7%] male; 20 559 [55·7%] White, 4842 [13·1%] Black or African American, 286 [0·8%] Native Hawaiian or other Pacific Islander, 300 [0·8%] Asian, 139 [0·4%] American Indian or Alaska Native, 524 (1·4%) other or mixed race, and 10 264 [27·8%] of unknown race). Clinical severity and instability were independent predictors of risk of hospitalisation (adjusted hazard ratio [HR] 1·09, 95% CI 1·07–1·10 for every SD increase in instability; 1·11, 1·09–1·12 for every SD increase in severity; p<0·0001 for both). These associations were consistent across all diagnoses, age groups, and in both males and females, as well as in several robustness analyses, including when clinical severity and clinical instability were based on the Patient Health Questionnaire-9 rather than Clinical Global Impression Severity measurements. Patients in the top half of the cohort for both clinical severity and instability were at an increased risk of hospitalisation compared with those in the bottom half along both dimensions (HR 1·45, 95% CI 1·39–1·52; p<0·0001).

**Interpretation:**

Clinical instability and severity are independent predictors of future risk of hospitalisation, across diagnoses, age groups, and in both males and females. These findings could help clinicians make prognoses and screen patients who are most likely to benefit from intensive interventions, as well as help health-care providers plan service provisions by adding additional detail to risk prediction tools that incorporate other risk factors.

**Funding:**

National Institute for Health and Care Research, National Institute for Health and Care Research Oxford Health Biomedical Research Centre, Medical Research Council, Academy of Medical Sciences, and Holmusk.

## Introduction

Although sometimes necessary for people with severe mental illness, admission to a psychiatric hospital is a costly^1^ and restrictive treatment option. It can sometimes have negative medical consequences by disrupting trust and transparency in the doctor–patient relationship.^2^ It can also have negative social consequences by interrupting career progression and limiting career prospects due to stigmatisation.^3^ Children and adolescents admitted to psychiatric hospitals might miss out on educational opportunities, which could contribute to social exclusion.^4^

For these reasons, psychiatric admission is often seen as a last resort and considerable efforts are made to try and prevent it when possible. For example, crisis resolution and home treatment teams have been created in the UK, which provide 24 h access and intensive support specifically to prevent admission. Although effective,^5^ these strategies are costly^6^ and are therefore reserved for patients at high risk of hospitalisation.

Predicting the risk of hospitalisation is thus crucial to identifying individuals most likely to benefit from intensive community care. Even when hospitalisation cannot be prevented, forecasting its risk would help in the planning of service provisions (eg, to manage bed availability). Finally, it would accelerate research in interventions aimed at preventing hospitalisation by enabling recruitment of patients at higher risk of the outcome and thus increasing statistical power.

**Research in context**

**Evidence before this study**

We searched PubMed (Medline) on Aug 23, 2022, for publications since database inception with no language restriction (provided the title was translated into English) using the terms “Predict*[Title] AND (Hospital*[Title] OR Inpatient[Title] OR admi*[Title] OR readmi*[Title]) AND (mental[Title] OR psych*[Title] OR depress*[Title] OR bipolar[Title] OR anxiety[Title] OR post-trauma*[Title] OR PTSD[Title] OR schiz*[Title] OR attention[Title] OR ADHD[Title] OR personality[Title] OR dementia[Title])”. We identified several studies that sought to predict psychiatric hospitalisation but these were limited to a specific diagnosis (eg, schizophrenia) or a specific clinical scenario (eg, patients in crisis). We found no study seeking transdiagnostic predictors of psychiatric hospitalisation unrestricted to a specific clinical scenario.

**Added value of this study**

To our knowledge, this is the first study to assess whether early clinical trajectory is a predictor of later risk of psychiatric admission across psychiatric diagnoses. We operationalised clinical severity and clinical instability using time series of Clinical Global Impression Severity scores, and found that both severity and instability early in a patient's trajectory (ie, during the first 2 months of clinical encounters) are independently associated with a significantly higher risk of psychiatric hospitalisation within the following 6 months. This finding was the case across a wide range of diagnoses, for both children (ie, those <18 years old) and adults (ie, those ≥18 years old), and both females and males.

**Implications of all the available evidence**

Clinical instability can help to predict future risk of psychiatric hospitalisation across diagnoses. These findings have implications for clinical practice (in which instability in Clinical Global Impression Severity trajectories can be used when making prognosis or targeting intensive interventions), planning of service provision (for which they can be used to predict the needs for hospital beds), and research (for which they can help in the design of studies testing interventions aimed at decreasing the risk of hospitalisation and to improve predictive models of hospitalisation).

Several studies have attempted to identify predictors of hospitalisation.^7^, ^8^, ^9^, ^10^, ^11^, ^12^, ^13^ However, they focus on specific diagnoses (eg first-episode psychosis,^7^ schizophrenia,^9^, ^10^ depression,^13^ or children with manic symptoms^12^) or clinical scenarios (eg, post-partum period^11^ or emergency assessments^8^). In addition, many of these studies are based on clinical trial data (which might not be routinely available), and the recruitment of trial participants (which limits generalisability).^7^, ^9^, ^10^

In this study, we hypothesised that clinical instability is a transdiagnostic risk factor for hospitalisation that can be measured in clinical practice. The motivations behind this hypothesis are three-fold. First, it has precedents in other fields of medicine (such as the observation that instability in glucose concentrations can lead to worse outcomes than chronically raised concentrations).^14^ Second, in psychiatry, mood instability is known to be associated with worse outcomes even in non-affective disorders,^15^ which might reflect a more general association between unstable illness and worse prognosis. Third, instability in any system is known to predict extreme events^16^ (appendix pp 1–7). We aimed to test this hypothesis by operationalising clinical instability using time series of Clinical Global Impression Severity (CGI-S) measurements and assessing whether clinical instability measured early in a patient's clinical trajectory is a predictor of later psychiatric hospitalisation.

## Methods

### Data sources

This study leveraged deidentified data derived from electronic health records, from the NeuroBlu Database (NeuroDB; Holmusk Technologies, New York, NY, USA), a longitudinal database comprising patient-level clinical data from 25 mental health centres across the USA spanning 20 years. Mental health centres provide secondary psychiatric care in both inpatient and outpatient settings. The primary data came from sites using the MindLinc electronic health records system accessed via NeuroDB release 21R1, which is described in a published cohort profile.^17^ More than 80% of the patients in this database have measurements of CGI-S: a 7-point measurement of illness severity,^18^ often used across diagnoses.

Structured data for sociodemographic factors, diagnosis (ie, ICD-9 and ICD-10 codes), and longitudinal CGI-S scores were available (all recorded between March, 1999, and December, 2020). Querying NeuroDB and analysing the data is achieved via a secure web-based interface with R code engine.

The NeuroDB platform has received a waiver of authorisation for analysis of deidentified health-care data from the WCG Institutional Review Board (reference: WCG-IRB 1-1470336-1). As a waiver of authorisation was obtained, ethical approval and individual patient consent were not required to conduct this study. This study followed the TRANSD recommendations^19^ (when applicable) and the Reporting of studies Conducted using Observational Routinely-collected Data guidelines (appendix pp 16–24).^20^

### Cohort definition

Our study included patients with an ICD-9 or ICD-10 code of major depressive disorder, bipolar disorder, generalised anxiety disorder, post-traumatic stress disorder (PTSD), schizophrenia or schizoaffective disorder, ADHD, or personality disorder. Diagnoses were preselected to represent a range of categories of psychiatric disorders (including mood, anxiety, psychotic, neurodevelopmental, and personality disorders) that can lead to hospitalisation. The complete list of codes can be found in the appendix (pp 8–9). Diagnoses were not mutually exclusive; each diagnosis was represented with a binary variable (present *vs* absent) in the statistical model.

To be included, patients had to have their first five CGI-S measurements recorded within a 2-month period, which we refer to as the phenotyping period. All patients who had been hospitalised before or within the phenotyping period were excluded (appendix p 15).

### Exposure of interest

A simple measure of the instability of a time series is the root mean square of successive differences (RMSSD). The RMSSD is widely used to measure time series instability, in particular in psychiatry.^21^ The RMSSD of clinical scores on mood disorder questionnaires can help differentiate patients with bipolar disorder, borderline personality disorder, and healthy controls^22^, ^23^ and to correlate with home and work functioning.^24^

The conventional RMSSD assumes that all recorded values are equally separated in time, which is often not the case in data from electronic health records for which the time between subsequent clinical encounters might vary. We therefore generalised the RMSSD to account for differences in intervals between subsequent CGI-S measurements as follows:

$$\text{tRMSSD}=\sqrt{\frac{\text{1}}{\text{N}}\sum_{i=1}^{N-1}{\left(\frac{C_{i+1}-C_{i}}{t_{i+1}-t_{i}}\right)}^{2}}$$

where *C*_i_ (*i=1,…, N*) are the *N* measurements of CGI-S and *t*_i_ (*i=1,…,N*) are the timestamps at which measurements were recorded, so that *t*_i+1_*–t*_i_ is the difference in time between two subsequent measurements. This generalisation results in the original RMSSD if all *t*_i+1_*–t*_i_ are equal. A detailed introduction to the concept and operationalisation of clinical instability is provided in the appendix (pp 1–7).

The mean CGI-S score during the phenotyping period was also calculated. This score served both as a covariate (to consider whether differences in tRMSSD reflect differences in mean CGI-S) and as a benchmark for effect sizes (because the mean CGI-S is anticipated to be strongly correlated with risk of hospitalisation). We refer to the tRMSSD of CGI-S during the phenotyping period as clinical instability and the mean CGI-S during the same period as clinical severity.

### Outcomes

The primary outcome was any inpatient stay as coded using the Observational Medical Outcomes Partnership Common Data Model^17^ within 6 months after the end of the 2-months phenotyping period. This duration of follow-up matches that used in a clinical trial showing the effectiveness of a crisis resolution team at preventing hospitalisation.^5^ For the time-to-event analysis, patients who had no hospitalisation were censored at the time of their last visit or at 6 months, whichever came first.

### Statistical analysis

For the time-to-event analysis, the Cox proportional hazard model was used. The null hypothesis that coefficients of the Cox model were equal to 0 (ie, adjusted hazard ratio [HR] equal to 1) was tested using a log-rank test. The proportional hazard assumption for the association between mood instability and hospitalisation in the primary analysis was tested with the generalised Schoenfeld approach^25^ and rejected if p was less than 0·05.

The analysis was adjusted for gender (as recorded in participants' health records), number of years in education, age, race (defined as White, Black or African American, Native Hawaiian or other, Pacific Islander, Asian, American Indian or Alaska Native, other or mixed race, or unknown), and psychiatric diagnosis. There were no data missing for age and years in education. Unknown gender and unknown race were considered as separate categories, alongside the other categories. Continuous covariates, clinical severity, and clinical instability were standardised (ie, transformed to a Z score). HRs are reported for the risk of hospitalisation during the follow-up period associated with 1 SD difference in the predictor variable (ie, a one unit difference in Z score-normalised values). Statistical significance was set at a two-tailed p value of less than 0·05.

There is no straightforward formula to link differences in RMSSD to statistical power. Instead, we relied on a recently developed empirical framework^26^ to assess statistical power (using affective trajectories as proxies for CGI-S trajectories). With a minimum group size of 3594 (representing the smallest subgroup in our study), to reach a statistical power of 90% to detect a small effect (1% of the variance) with an alpha of 0·05, five observations per individual are enough to calculate the RMSSD.

We performed several secondary analyses to assess the specificity and robustness of our results. First, an unadjusted analysis was conducted to assess whether covariates substantially affected the results. Second, we analysed whether the association between clinical instability and risk of hospitalisation held across diagnoses by stratifying the dataset into different diagnoses. Third, we stratified the cohort between children (ie, those <18 years old) and adults (ie, those ≥18 years old) and between female and male genders to assess whether the results held across demographic subgroups.

We subsequently assessed the robustness of the results to several changes in model and population specification. First, we expanded the follow-up window from 6 months to 1 year. Second, we expanded the phenotyping window from 2 months to 6 months while keeping the minimum number of CGI-S measurements constant, thus broadening the eligible population and assessing the generalisability of the findings. Third, we included the number of CGI-S measurements available during the phenotyping window as a covariate (testing whether higher clinical instability merely reflects more frequent visits). Fourth, we included the date of the first CGI-S measurement (in yearly bins) as a categorical covariate to encode possible confounding by changes in clinical practice over time. Fifth, the population was limited to people with no missing data (ie, race and gender were known). Sixth, the analysis was replicated using a different cohort of patients with the time series of Patient Health Questionnaire-9 (PHQ-9; rather than CGI-S) measurements used to calculate clinical severity and instability (appendix pp 7–8). This allowed assessment of the generalisability of the findings and complemented the primary analysis, as PHQ-9 does not depend on subjective assessments of illness severity by a clinician. Because the frequency of PHQ-9 scores was substantially lower than that for CGI-S, we used a 1-year phenotyping window with at least four PHQ-9 measurements in this analysis. Years of education was not available for this cohort.

Finally, we explored whether clinical instability and severity were independent predictors of risk of hospitalisation in three ways. First, we estimated the HR for each variable using a Cox model not adjusted for the other (but still adjusted for other covariates). If instability and severity were collinear, then the HR would typically increase when the other variable was removed. Second, we added an interaction term between instability and severity in the Cox model. The absence of a significant interaction would support the independence of the two predictors. Third, we stratified the population into quadrants based on median splits along clinical severity and instability, with those having low clinical severity and low clinical instability taken as a reference. This grouping allowed assessment of the interaction between clinical severity and instability beyond the linear assumption of the Cox model with continuous measurements.

### Role of the funding source

The funders of the study had no role in the study design, data analysis, data interpretation, writing of the manuscript, or decision to publish the study findings.

## Results

36 914 patients met the inclusion criteria (table 1; appendix p 12). The median follow-up time was 180 days (IQR 101–180). The prevalence of each psychiatric diagnosis within the cohort varied from 9·7% (for schizophrenia or schizoaffective disorder) to 42·8% (for major depressive disorder).

**Table 1:** Baseline characteristics

		**Patients (N=36 914)**
Gender		
	Female	21 156 (57·3%)
	Male	15 748 (42·7%)
	Not recorded	10 (<0·1%)
Race		
	White	20 559 (55·7%)
	Black or African American	4842 (13·1%)
	Native Hawaiian or Other Pacific Islander	286 (0·8%)
	Asian	300 (0·8%)
	American Indian or Alaska Native	139 (0·4%)
	Other or mixed race	524 (1·4%)
	Unknown	10 264 (27·8%)
Age, years		29·7 (17·5)
Years of education		4·51 (6·75)
Diagnosis		
	Major depressive disorder	15 786 (42·8%)
	Bipolar disorder	8621 (23·4%)
	Generalised anxiety disorder	7069 (19·1%)
	Post-traumatic stress disorder	10 966 (29·7%)
	Schizophrenia or schizoaffective disorder	3594 (9·7%)
	ADHD	9229 (25·0%)
	Personality disorder	5866 (15·9%)
Follow-up time, days		180 (101–180)
Number of Clinical Global Impression-Severity measurements for phenotyping		7 (6–10)

Data are n (%) or mean (SD) or median (IQR).

**Figure 1:**
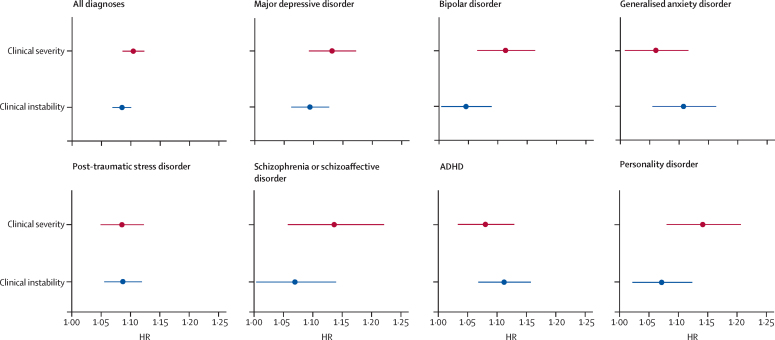
HRs for the 6-month risk of hospitalisation corresponding to an increase by 1 SD in clinical instability and clinical severity The horizontal bars represent 95% CIs. The results are given for the primary model with all diagnoses included as well as for each diagnosis independently. HR=hazard ratio.

Higher clinical instability during the phenotyping period was associated with a significantly higher risk of hospitalisation in the following 6 months (HR for every 1 SD increase in clinical instability 1·09, 95% CI 1·07–1·10, p<0·0001; figure 1). There was no violation of the proportionality assumption (χ^2^=0·62; p=0·43). The association was of similar magnitude to that between clinical severity and risk of future hospitalisation (HR for every 1 SD increase in clinical severity: 1·11, 95% CI 1·09–1·12, p<0·0001; figure 1).

An association of both clinical severity and clinical instability with risk of hospitalisation was observed across diagnoses (figure 1, table 2). For each diagnosis, higher clinical instability was significantly associated with increased risk of hospitalisation within the next 6 months (HRs for every 1 SD increase in clinical instability: 1·05–1·11, all p<0·05). The association was strongest among patients with ADHD (1·11, 1·07–1·16; p<0·0001) and generalised anxiety disorder (1·11, 1·07–1·16; p<0·0001). The association was weaker for bipolar disorder (1·05, 95% CI 1·01–1·09, p=0·025) and schizophrenia or schizoaffective disorder (1·07, 1·00–1·14, p=0·038).

**Table 2:** Results of the primary and secondary analyses

		**Clinical instability**		**Clinical severity**		**Hospitalisations**	
		HR (95% CI)	p value	HR (95% CI)	p value	Cohort size	Number of events (%)
Primary analysis, fully adjusted*		1·09 (1·07–1·10)	<0·0001	1·11 (1·09–1·12)	<0·0001	36 914	9294 (25·2%)
Analysis adjusted for sociodemographic factors†		1·07 (1·05–1·09)	<0·0001	1·09 (1·07–1·11)	<0·0001	36 914	9294 (25·2%)
Unadjusted analysis		1·07 (1·06–1·09)	<0·0001	1·11 (1·09–1·12)	<0·0001	36 914	9294 (25·2%)
Individual diagnoses							
	Major depressive disorder	1·09 (1·06–1·12)	<0·0001	1·13 (1·09–1·17)	<0·0001	15 786	3814 (24·2%)
	Bipolar disorder	1·05 (1·01–1·09)	0·025	1·12 (1·07–1·17)	<0·0001	8621	2096 (24·3%)
	Generalised anxiety disorder	1·11 (1·06–1·17)	<0·0001	1·06 (1·01–1·12)	0·018	7069	1773 (25·1%)
	Post-traumatic stress disorder	1·09 (1·06–1·12)	<0·0001	1·09 (1·05–1·12)	<0·0001	10 966	3311 (30·2%)
	Schizophrenia or schizoaffective disorder	1·07 (1·00–1·14)	0·038	1·14 (1·06–1·22)	0·00037	3594	716 (19·9%)
	ADHD	1·11 (1·07–1·16)	<0·0001	1·08 (1·04–1·13)	0·00035	9229	2235 (24·2%)
	Personality disorder	1·07 (1·03–1·13)	0·0026	1·14 (1·08–1·21)	<0·0001	5866	1473 (25·1%)
Age groups							
	Adults (≥18 years old)	1·10 (1·08–1·12)	<0·0001	1·15 (1·12–1·17)	<0·0001	23 829	5333 (22·4%)
	Children (<18 years old)	1·05 (1·03–1·08)	<0·0001	1·05 (1·02–1·08)	0·00037	13 085	3961 (30·3%)
Gender							
	Female	1·09 (1·07–1·11)	<0·0001	1·13 (1·11–1·16)	<0·0001	21 156	5254 (24·8%)
	Male	1·08 (1·06–1·10)	<0·0001	1·08 (1·05–1·10)	<0·0001	15 748	4039 (25·6%)
Other robustness analyses							
	1-year follow up	1·06 (1·05–1·08)	<0·0001	1·09 (1·07–1·10)	<0·0001	36 914	13 922 (37·7%)
	Number of CGI-S measurements included as a covariate	1·06 (1·05–1·08)	<0·0001	1·10 (1·08–1·12)	<0·0001	36 914	9294 (25·2%)
	Longer phenotyping window	1·19 (1·18–1·21)	<0·0001	1·23 (1·21–1·24)	<0·0001	70 304	14 903 (21·2%)
	Adjusted for year of first CGI-S measurement	1·06 (1·05–1·08)	<0·0001	1·08 (1·06–1·10)	<0·0001	36 914	9294 (25·2%)
	Complete cases only	1·09 (1·07–1·10)	<0·0001	1·15 (1·13–1·17)	<0·0001	26 648	6885 (25·8%)
	Clinical instability and clinical severity measured with the Patient Health Questionnaire-9 instead of the CGI-S	1·11 (1·02–1·22)	0·019	1·14 (1·01–1·28)	0·033	4557	149 (3·3%)

HRs, 95% CIs, and p values are provided for clinical instability and clinical severity as predictors of hospitalisation. The cohort size and number of events are also provided. HR=hazard ratio. CGI-S=Clinical Global Impression-Severity.

* Adjusted for gender, number of years in education, age, race, psychiatric diagnosis, and for the other predictor (ie, the analysis of clinical instability was adjusted for clinical severity and vice versa).

† Adjusted for gender, number of years in education, age, race, and psychiatric diagnosis.

The association between clinical instability and future risk of hospitalisation was found to be robust in various scenarios (table 2). It was significant in an unadjusted model, in adults (≥18 years old) and children (<18 years old), and in females and males. Clinical instability was also a significant predictor of hospitalisation up to 1 year later. The association was robust to adjustment for the number of CGI-S measurements during the 2-month phenotyping period, to adjustment for the year of first CGI-S measurement, and to restriction of the cohort to people without missing data. It was found to generalise to a larger population of people with five or more CGI-S measured over a 6-month (rather than 2-month) phenotyping period (appendix p 13). Finally, it was also found to generalise to a cohort in whom clinical severity and instability were defined based on PHQ-9 (rather than CGI-S) measurements (appendix p 14). Across all scenarios, the association between clinical instability and future hospitalisation was of comparable magnitude to that between clinical severity and future hospitalisation (table 2).

**Figure 2:**
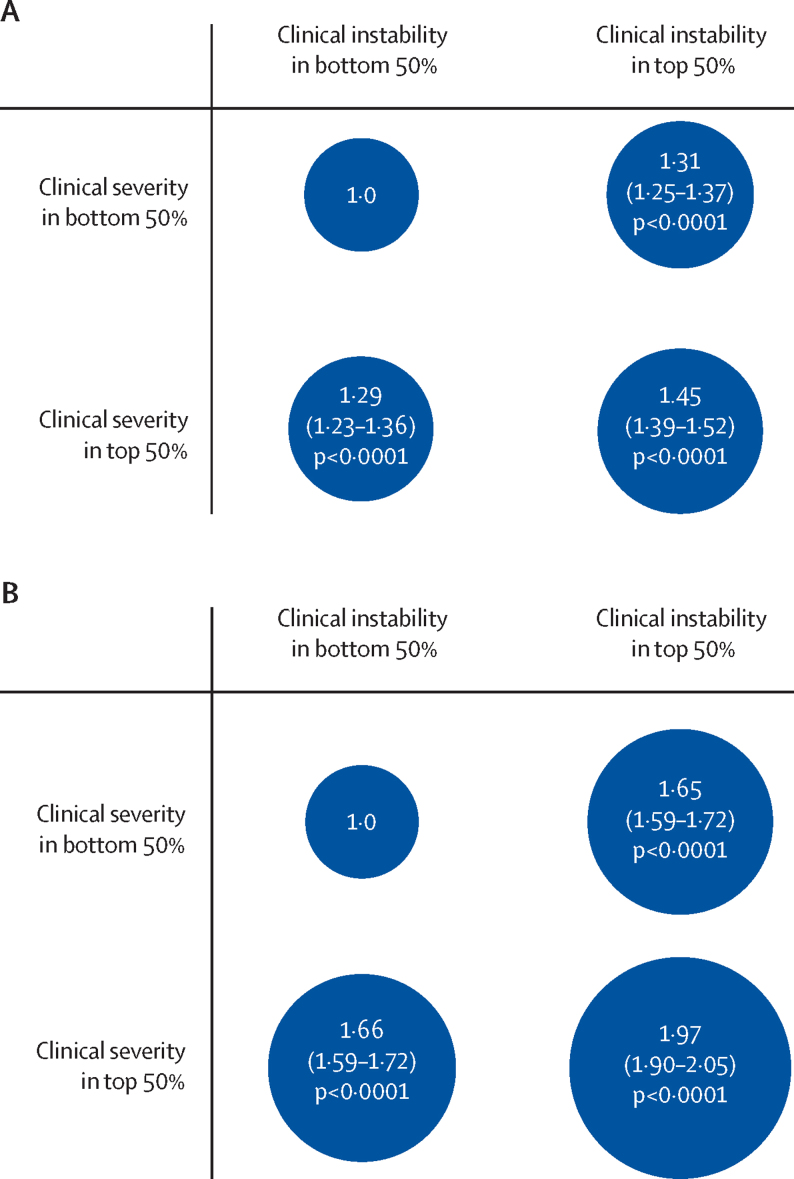
HR for the risk of being hospitalised for those in the top versus bottom half of the population in terms of clinical severity and clinical instability (A) within the primary analysis, and (B) in the analysis with a 6-months phenotyping window Each comparison is made with respect to the reference taken to be the subgroup in the bottom half of the population both in terms of severity and instability. The diameter of each circle is proportional to the HR, which is reported alongside its 95% CI within each circle. The total number of individuals and hospitalisations in each subgroup is provided in the appendix (p 12). HR=hazard ratio.

Clinical instability and clinical severity were largely independent predictors of future risk of hospitalisation. There was no interaction between the two (HR 0·99, 95% CI 0·98–1·01; p=0·33) and the HR of one did not inflate when the other was removed from the model (1·07, 1·05–1·09 for instability, and 1·09, 1·07–1·11 for severity). In addition, compared with those in the bottom half of the population both in terms of clinical severity (median 4·0) and instability (median 0·066), those in the top half in terms of both severity and instability were at an increased risk of hospitalisation within 6 months (HR 1·45, 95% CI 1·39–1·52; p<0·0001; figure 2) whereas those in the top half in terms of either severity or instability had lower HRs. A similar pattern was observed along the four quadrants when the phenotyping period was extended to 6 months with a risk of hospitalisation that almost doubled for those in the top half of the population in terms of both clinical severity and clinical instability compared with those in the bottom half along those two dimensions.

## Discussion

Our study, based on data from electronic health records from 36 914 patients in secondary mental health care, found that clinical severity and clinical instability are independent and robust predictors of future risk of hospitalisation. This finding persisted across diagnoses, age groups, and genders. The magnitude of the association was clinically significant. An HR of 1·09 for every SD difference in clinical instability implies HRs of 1·19 or 1·30 when comparing people who are 2 or 3 SDs apart; and HRs of greater magnitude were observed for a more general population in whom the phenotyping period was increased to 6 months.

Individuals in the top half of the population in terms of both clinical severity and clinical instability were at a 45% (95% CI 39–52) increased risk of hospitalisation within the next 6 months compared with those in the bottom half of the population along these two dimensions. To illustrate how this increased hospitalisation risk translates in practical terms, let us assume that we have an intervention that reduces the risk of hospitalisation by 80% (eg, implementation of a crisis resolution team compared with treatment as usual),^5^ and a population of 1000 patients in crisis with a 50% risk of hospitalisation without intervention. And let us assume that the services covering this population have the capacity to apply the intervention to only 250 of the 1000 patients. Applying the intervention indiscriminately to the population (ie, selecting 250 patients at random) would result in 400 hospital admissions (100 admissions prevented by the intervention). If our findings apply to this population, then applying the intervention to patients selected among those within the top half of the population in terms of both clinical severity and clinical instability would result in 385 patients being hospitalised (115 admissions prevented by the intervention). In other words, by selecting patients on the ground of their clinical severity and clinical instability and without increasing service provision, an additional 15 admissions could be prevented in this simplistic scenario. The detailed calculations for this illustration are provided in the appendix (pp 10–11).

This example is based on many assumptions that might not be valid in practice (eg, our findings might not strictly apply to a population of patients at high risk of hospitalisation, and within this population, clinicians might use other information to inform their decisions to intervene). More research is needed to assess whether and how our findings would apply to such scenarios. In addition, predictive models typically combine multiple features to improve the sensitivity and specificity of the prediction. This study showed the relevance and effect of clinical instability as a simple and transdiagnostic risk factor, which could be integrated into more elaborate predictive models.

Another potential application of the ability to predict the risk of hospitalisation is in recruiting patients in clinical trials of new treatments. If hospitalisation is chosen as a hard outcome in such studies, then selecting a cohort with a higher baseline risk of hospitalisation might increase statistical power, which can result in studies that are more cost-effective.

Besides these translational potentials, our findings could provide insights into the dynamic nature of psychiatric illness. The association between higher instability in global impression and poorer outcomes is compatible with the conceptualisation of mental illnesses as dynamic systems, akin to the observation that unstable dynamics of mood variability could signal the onset of depression.^27^ But CGI-S does not only capture the patient's disease severity, it also captures the clinician's perception thereof. High CGI-S instability could, in part, reflect a clinician's uncertainty in addition to a truly unstable mental state (even though the replication of our findings based on PHQ-9 measurements suggests that clinician's uncertainty is unlikely to account for the whole association). Therefore, the current findings might in part be manifestations of an association between clinical uncertainty and poorer prognosis. Such an association would come as no surprise to practising clinicians, but its magnitude would be relevant for clinical practice. It would clarify and highlight the importance for clinicians to acknowledge their uncertainty and leverage this awareness when making prognoses.

This study has several strengths including a large sample size, inclusion of a diversity of diagnoses and age groups, the use of routinely collected data across multiple health centres (thus increasing translational potential), and the robustness of the findings across secondary analyses. However, it also has several limitations. First, we did not separately assess voluntary versus compulsory admission. Second, the study focused on first admissions rather than recurrent admissions. This outcome was thought to be most valuable because previous hospitalisation tends to trump other predictors of future hospitalisation.^7^ Third, although many of the most common diagnoses were included in our study, others (eg, dementia) were not included because too few patients were identified in the database. Fourth, although we showed the predictive value of CGI-S trajectories, this study cannot explain why more unstable trajectories are associated with higher risk of hospitalisation. Although several potential explanations are outlined in this Article, further studies are needed to understand these relationships. In particular, the simplicity of CGI-S as a single-item global measure is its strength for repeated measurements in clinical settings but is also its weakness as it is unclear which aspects of psychopathology it measures. The replication of our findings using PHQ-9 measurements sought to address this limitation and larger-scale replications using such multidimensional scales are needed. Fifth, because this study is observational, one cannot infer that reducing clinical severity or clinical instability will necessarily lead to reduced risk of hospitalisation. Sixth, subgroup analyses assessed robustness of the results in different subgroups, but statistical analyses were not performed to assess whether significant heterogeneity was present.

In summary, clinical severity and clinical instability early in a patient's clinical journey are both robustly associated with a higher risk of future hospital admission. The definition and operationalisation of clinical instability using CGI-S records could serve as a transdiagnostic phenotype that provides information complementary to clinical severity and could improve outcome prediction in clinical practice. These findings can help develop prognostic models, which might be useful to identify patients most likely to benefit from intensive community interventions, plan service provision, recruit patients into studies, and gain insights into the dynamic nature of psychopathology.

## Data sharing

Data can be obtained from a third party and are not publicly available. Deidentified data can be analysed through the NeuroBlu platform. Please e-mail info@neuroblu.ai for further information on accessing NeuroBlu.

## Declaration of interests

MT has received consultancy fees from Holmusk, which developed NeuroBlu, and is a clinical advisor to Akrivia Health. RP has received personal fees from Holmusk (consulting and employment), grant funding from Janssen, and honoraria from Boehringer Ingelheim. All other authors are current or former employees of Holmusk.
