# Supplementary material for: Early trajectory of clinical global impression as a transdiagnostic predictor of psychiatric hospitalisation: a retrospective cohort study
Source: Lancet Psychiatry. 2023 May;10(5):334–41. doi: 10.1016/S2215-0366(23)00066-4 (PMC10709150; doi:10.1016/S2215-0366(23)00066-4)
Supplement: Supplementary appendix [file mmc1.pdf]

THE LANCET
Psychiatry

Supplementary appendix

This appendix formed part of the original submission and has been peer reviewed. We post it as supplied by the authors.

Supplement to: Taquet M, Griffiths K, Palmer EOC, et al. Early trajectory of clinical global impression as a transdiagnostic predictor of psychiatric hospitalisation: a retrospective cohort study. *Lancet Psychiatry* 2023; published online March 23. https://doi.org/10.1016/S2215-0366(23)00066-4.

Table of Contents

Clinical instability: introduction and operationalization 1

Description of the network for the PHQ-9 data 7

Diagnostic codes 8

Translating the effect size into clinically relevant effect 10

Supplementary Tables 12

Other supplementary Figure 15

References 15

RECORD statement 16

Clinical instability: introduction and operationalization

<u>Introduction</u>

Any time series (i.e. sequence of consecutive measurements) can be summarized by a set of simple values (sometimes referred to as summary statistics). The mean of all values is one of the simplest summary statistics. If one is interested in how values are spread around the mean, then one might also calculate the standard deviation of the values. Sets of values that are not time series are often summarised simply by their mean and standard deviation which represent the coarse grain aspects of their distribution. However, these two summary statistics are often insufficient to represent even coarse-grain aspects of time series: time series with the same mean and standard deviation can look very different (Fig. S1).

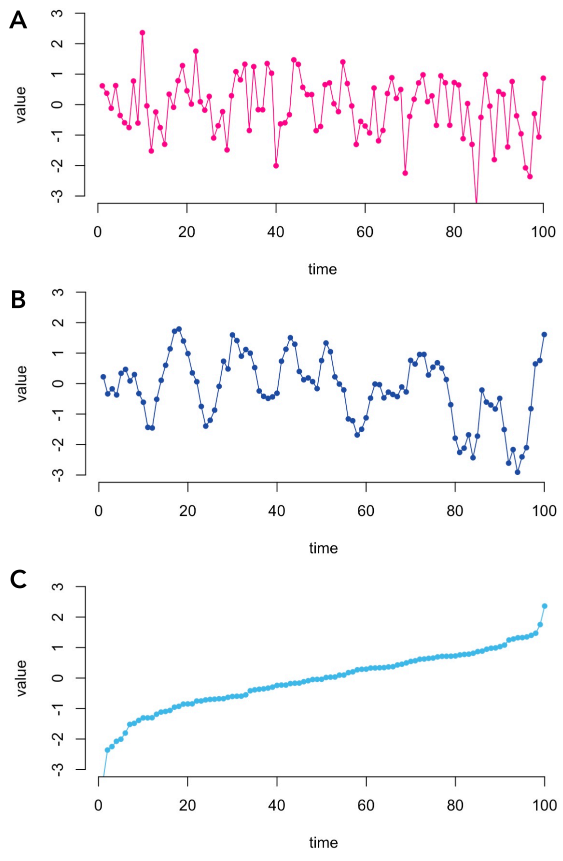

Fig. S1 – Example time series with the same mean (= 0) and standard deviation (= 1) but very different instability (1.41 in A, 0.59 in B, and 0.03 in C).

What differs in the three time series represented in Fig. S1 is how much they fluctuate: the one in Fig. S1A fluctuates frequently, that in Fig. S1B fluctuates occasionally, and the one in Fig. S1C does not fluctuate at all. We refer to the degree of fluctuation of a time series as its *instability*.[1] In the next subsection, we formalise how the idea of instability can be translated into a number (i.e. a summary statistic).

Operationalization

One way to start operationalizing the concept of instability introduced above is to notice that time series in Fig. S1 differ by how much the value can change from one time point to the next. In Fig. S1A, the value can go from one extreme to the other in a single step, whereas in Fig. S1B there are always intermediate steps between extreme values. We can therefore quantify the instability of the time series via the difference between two consecutive values. Denoting by C_i the *i*-th value of the time series, the difference with the subsequent value is $C_{i+1} - C_i$. Because we are interested in the magnitude of the difference but not its sign, we can focus on the squared differences: $(C_{i+1} - C_i)^2$. Averaging over all pairs of successive values gives us the mean square of successive differences and taking the square root of the result gives us the root mean square of successive differences (RMSSD):

$$\text{RMSSD} = \sqrt{\frac{1}{N}\sum_{i=1}^{N-1}(C_{i+1} - C_i)^2}.$$

This is the original definition of the RMSSD as seen in previous papers.[2–4] However, this definition requires one last adaptation. So far, we have assumed that values of the time series were measured at constant intervals (e.g. in Fig. S1, there is one value for every unit of time). In clinical practice where CGI-S is recorded during different clinical encounters, the intervals between subsequent recordings might vary. This is an important consideration because differences between values measured further apart in time might be larger and this might not

reflect faster fluctuations of the time series. An example of this is represented in Fig. S2 which shows both the original time series of Fig. S1B and a version of the same time series in which 30% of values have been removed at random. Because the overall shape of the curve is the same in the two scenarios, their instability should be the same.

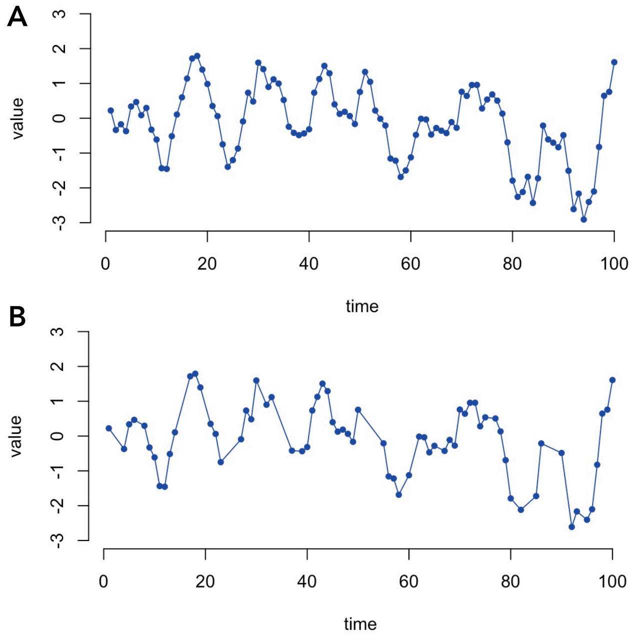

Fig. S2 – (A) Original time series presented in Fig. S1B. (B) The same time series after removing 30% of values at random. The overall shape of the time series is similar in the two scenarios and so their measure of instability should be similar. This is achieved by adjusting the RMSSD for time.

This can be achieved by adjusting the successive differences for time. If we denote by t_i the time at which the value C_i was recorded, then instead of focussing on the difference between subsequent values, we can focus on the rate at which the value changes with time: $\frac{C_{i+1}-C_i}{t_{i+1}-t_i}$.

Instead of averaging the squared differences, we can average the squared rates of change. Taking the square root of the result gives us the time-adjusted root mean square of successive differences (tRMSSD) which we used in this study:

$$\text{tRMSSD} = \sqrt{\frac{1}{N}\sum_{i=1}^{N-1}\left(\frac{C_{i+1} - C_i}{t_{i+1} - t_i}\right)^2}.$$

If a measurement is available for every unit of time, then $t_{i+1} - t_i = 1$ and tRMSSD is the same as RMSSD. Of note, the quantity $\frac{C_{i+1}-C_i}{t_{i+1}-t_i}$ can also be interpreted as the slope of the line that connects two subsequent values. This geometrical interpretation means that if unrecorded values lie on the line that connects measured values, then adding or removing them makes little difference to the tRMSSD.

Using this formula, we can now calculate the instability for the time series in Fig. S1: 1.41 in A, 0.59 in B, and 0.03 in C.

Code

The tRMSSD is easily calculated using R (or any other programming language for numeric calculation). Using as input a vector of measurement times (e.g. timestamps or dates) which we denote `times` and a vector of values measured at those times which we denote `values`, the following function in R calculates the tRMSSD:

```
tRMSSD <- function(times,values){
     n=length(values)
     dtime = times[2:n]-times[1:n-1]
     dvals = values[2:n]-values[1:n-1]
     res = sqrt(1/n*sum((dvals/dtime)^2))
     return(res)
}
```

Why might clinical instability predict hospitalization: a mathematical example

So far, we have seen what clinical instability is and how it can be operationalised. In this section, we provide an intuition for its association with later hospitalization. We use a simplistic mathematical model to illustrate the main point.

Let us assume that a patient's clinical state can be summarised by a single number with higher values representing higher severity. And let us assume that this value can change from day to day and that over a certain threshold, the patient requires hospitalization.

Now let us consider two groups of patients: group A and group B. Patients in group A have clinical states that are simply random numbers drawn every day from a Normal distribution with mean 0 and standard deviation 1. In mathematical terms, the clinical state C_i at time i is:

$$C_i = x_i, \quad \text{where } x_i \sim N(0,1).$$

To define the clinical states of patients in group B, we start with a sequence of random numbers x_i similar to group A, but this time, the clinical state at time i is a weighted average of the current value x_i and the previous 5 values $x_{i-1}, x_{i-2}, x_{i-3}, x_{i-4}, x_{i-5}$. In other words:

$$C_i = \frac{1}{\sqrt{6}}(x_i + x_{i-1} + x_{i-2} + x_{i-3} + x_{i-4} + x_{i-5}) \quad \text{where } x_i \sim N(0,1).$$

The coefficient $\frac{1}{\sqrt{6}}$ is used so that the standard deviation of the clinical state in group B is 1 (as in group A). So the clinical states in both group A and group B have the same mean (=0) and same standard deviation (=1). The difference between the two is that in group A, two

consecutive clinical states are independent from one another (i.e. knowing the clinical state at time *i* does not provide any information about the clinical state at time *i+1*). By contrast, in group B two consecutive clinical states are highly correlated because they share many of the terms in their definition.

The time series illustrated in Fig. S1 are actually examples of clinical states from group A (Fig S1A) and group B (Fig S1B) and we have already seen that instability in Fig. S1A is higher than in Fig. S1B. To assess whether the higher clinical instability in group A predicts higher risk of hospitalization, we can simulate 10,000 patients in each group, each having a time series of 1000 clinical states. Assuming that hospitalization is necessary (in this simplistic model) whenever the clinical state exceeds 3.5, we can then count the number of patients who meet this criterion in group A and group B. The results from this simulation are presented in Fig. S3. Significantly fewer patients in group B meet the ‘hospitalization’ criterion than in group A (1518 vs. 2064 out of 10,000, $Chi^2 = 101$, df=1, $p < 0.0001$).

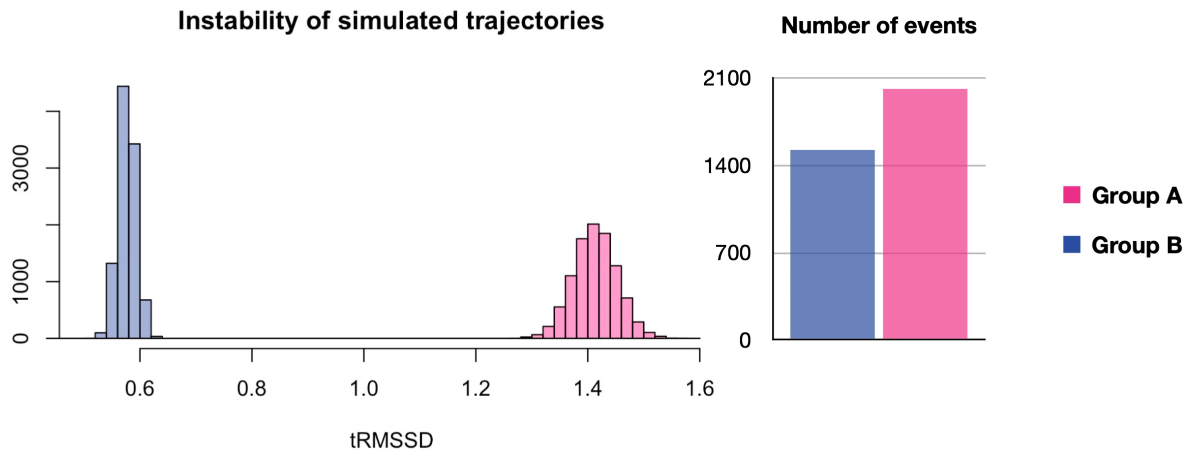

Fig. S3 –10,000 simulated trajectories in Group A and B with the same mean and standard deviation but different instability (left) show that those with lower instability have lower risk of ‘hospitalization’ (right).

The results from this simulation might at first seem remarkable. After all, each individual value of the clinical state follows the same distribution (a normal distribution with mean 0 and standard deviation 1) in group A and B. So why is it more likely to observe an extreme value in group A than it is in group B? The reason is that, in group A, every single day gives a new chance to observe an extreme value, whereas in group B values are correlated with the previous ones which decreases the risk of observing extreme values. In group A, an analogy would be the chance of observing at least one 6 when throwing two dices independently whereas in group B the two dices are first glued together so that they always display the same number. The chance of observing at least one 6 in the latter case is substantially lower (1 in 6 vs. 11 in 36).

Description of the network for the PHQ-9 data

The data for the primary analysis and all but one secondary analyses are based on NeuroDB 21R1 dataset which is fully described in a cohort profile paper.[5] The secondary analysis based on PHQ-9 data is based on a separate network of U.S. health care organizations which comprises the following:

1) A large behavioural and developmental disability care centre serving over 88,000 people per year. The centre serves a high percent of uninsured and Medicaid patients across their outpatient clinics that provide different mental health and substance use services. Though the centre provides outpatient services only, they keep records of patient hospitalization / transfer details with centre (2) where patients are often transferred to/from for inpatient services.

2) A large provider of inpatient psychiatric care, which serves a high percent of uninsured and Medicaid patients. This centre has some patients overlap with center (1) where patients are often transferred to/from for outpatient psychiatric services.

3) Specialty care clinics and neighbourhood-based health centres which serve a high percent of uninsured and Medicaid patients and handle more than 45,000 outpatient visits and 1,200 in-patient admissions annually. Some of the specialties include trauma care, cancer care, women's health, primary care and behavioural health.

4) A large provider of mental health services serving over 55,000 adults and children annually. Additionally, the centre provides primary care and serves homeless adults, youth, and families who have a mental health and/or a substance abuse challenge. The centre offers care through outpatient facilities that receives patients across 4 different neighbourhood hospitals and has a high percent of uninsured and Medicaid patients.

Diagnostic codes

The following diagnostic codes were used to define diagnoses in the NeuroBlu data.

Major Depressive Disorder (MDD):

ICD-9 codes : '296.20', '296.21', '296.22', '296.23', '296.24', '296.25', '296.26', '296.30', '296.31', '296.32', '296.33', '296.34', '296.35', '296.36'

ICD-10 codes : 'F32.0', 'F32.1', 'F32.2', 'F32.3', 'F32.4', 'F32.5', 'F32.81', 'F32.89', 'F32.9', 'F33.0', 'F33.1', 'F33.2', 'F33.3', 'F33.40', 'F33.41', 'F33.42', 'F33.8', 'F33.9'

Bipolar disorder (BD) :

ICD-9 codes : '296.40', '296.41', '296.42', '296.43', '296.44', '296.45', '296.46', '296.50', '296.51', '296.52', '296.53', '296.54', '296.55', '296.56', '296.60', '296.61', '296.62', '296.63', '296.64', '296.65', '296.66', '296.7', '296.80', '296.89'

ICD-10 codes : 'F31.0', 'F31.10', 'F31.11', 'F31.12', 'F31.13', 'F31.2', 'F31.30', 'F31.31', 'F31.32', 'F31.4', 'F31.5', 'F31.60', 'F31.61', 'F31.62', 'F31.63', 'F31.64', 'F31.70', 'F31.71', 'F31.72', 'F31.73', 'F31.74', 'F31.75', 'F31.76', 'F31.77', 'F31.78', 'F31.81', 'F31.89', 'F31.9'

Generalised Anxiety Disorder (GAD) :

ICD-9 code : ‘300.02’

ICD-10 code : ‘F41.1’

Post-Traumatic Stress Disorder (PTSD) :

ICD-9 codes : '309.81'

ICD-10 codes: 'F43.10', 'F43.11', 'F43.12'

Schizophrenia/Schizoaffective disorder (SCZ) :

ICD-9 codes : '295.00', '295.01', '295.02', '295.03', '295.04', '295.05', '295.10', '295.11', '295.12', '295.13', '295.14', '295.15', '295.20', '295.21', '295.22', '295.23', '295.24', '295.25', '295.30', '295.31', '295.32', '295.33', '295.34', '295.35', '295.40', '295.41', '295.42', '295.43', '295.44', '295.45', '295.50', '295.51', '295.52', '295.53', '295.54', '295.55', '295.60', '295.61', '295.62', '295.63', '295.64', '295.65', '295.80', '295.81', '295.82', '295.83', '295.84', '295.85', '295.90', '295.91', '295.92', '295.93', '295.94', '295.95', ‘295.7’, ‘295.7x’

ICD-10 codes : 'F20.0', 'F20.1', 'F20.2', 'F20.3', 'F20.5', 'F20.81', 'F20.89', 'F20.9', ‘F25’

Attention Deficit Hyperactivity Disorder (ADHD) :

ICD-9 codes : '314.00', '314.01'

ICD-10 codes : 'F90.0', 'F90.1', 'F90.2', 'F90.8', 'F90.9'

Personality Disorder (PD) :

ICD-9 codes : '301.0', '301.10', '301.11', '301.12', '301.13', '301.20', '301.21', '301.22', '301.3', '301.4', '301.50', '301.51', '301.59', '301.6', '301.7', '301.81', '301.82', '301.83', '301.84', '301.89', '301.9'

ICD-10 codes : 'F60.0', 'F60.1', 'F60.2', 'F60.3', 'F60.4', 'F60.5', 'F60.6', 'F60.7', 'F60.81', 'F60.89', 'F60.9'

Translating the effect size into clinically relevant effect

In the discussion, we illustrate how the effect size might translate into lower admission rates. Here we provide the details of how we calculated these.

We let:

- *E* be the effectiveness of the intervention, i.e. the reduction in probability of hospitalization for a patient who would have otherwise been admitted had they not received the intervention. We set *E*=80% in our example, to corresponds to the effectiveness of crisis resolution teams.
- *B* be the baseline admission rate in a population. We set *B*=50% to correspond to a population of patients in crisis.
- *C* the capacity of the service, i.e. the proportion of patients who can receive the intervention. We set *C*=25% in our example.

From a population of 1000 people, if we were to apply the intervention at random, we would select 250 patients randomly (= C*1000). Out of those 250 patients, 125 would have been hospitalized if they had not received the intervention (=B*250). However, because the intervention is effective, only 25 (=125*(1-E)) will be hospitalized. This is why we claimed that in this simple example, 100 admissions would have been prevented by the intervention.

Now let's assume that instead of applying the intervention at random, we apply it to the top half of the population in terms of clinical severity and instability (i.e. the 25% of the population at higher risk of hospitalization). In that population, separated in quadrants (and assuming that 25% of the population falls in each quadrant), the risk of hospitalization are as

follows (these can be derived from simple algebra to reach a total risk of 50% in the population while reflecting the hazard ratios seen in Fig. 3a in the paper):

- Bottom half in terms of both clinical severity and instability: 39.6%
- Bottom half in terms of severity, but top half in terms of instability: 51.1% (=39.6%*1.29)
- Top half in terms of severity, but bottom half in terms of instability: 51.9% (=39.6%*1.31)
- Top half in terms of both severity and instability: 57.4% (=39.6% * 1.45).

Within the latter group, there would be 144 admissions without the intervention (=57.4%*250). However, with the intervention, this number reduces to 29 (=144*(1-E)), hence 115 admissions have been prevented by the intervention (=144-29), or 15 more than if the intervention was applied at random.

Supplementary Tables

Table S1 – Association between data missingness and exposure/outcome

	Individuals with complete data	Individuals with missing data	SMD
Clinical severity, mean (SD)	4.14 (1.03)	3.97 (0.99)	0.17
Clinical instability, mean (SD)	0.19 (0.32)	0.21 (0.36)	0.083
Proportion of events, %	25.8	23.5	0.055

Abbreviations: SD, standard deviation; SMD, standardized mean difference

Table S2 – Summary statistics for the distribution of clinical severity in the whole cohort (i.e. marginal distribution), and those with clinical instability in the bottom and top half of the population in terms of clinical instability. The distributions are very similar indicating that a wide range of clinical severity can be observed for different levels of clinical instability.

	Whole cohort	Individuals with low clinical instability	Individuals with high clinical instability
Min	1.0	1.0	1.05
1st quartile	3.6	4.0	3.4
Median	4.0	4.1	4.0
Mean	4.1	4.3	3.9
3rd quartile	4.9	5.0	4.6
Max	7.0	7.0	6.9

Table S3 – Number of individuals and number of events in the analyses where clinical severity and instability are dichotomised.

	2-months measurement window		6-months measurement window	
	Number of individuals	Number of hospitalisations, n (%)	Number of individuals	Number of hospitalisations, n (%)
Low severity and low instability	9591	1917 (19.99)	20394	2829 (13.87)
Low severity and high instability	9797	2587 (26.41)	18096	4093 (22.62)
High severity and low instability	8963	2356 (26.29)	14758	4093 (22.62)
High severity and high instability	8563	2434 (28.42)	17056	4577 (26.84)

Table S4 - Baseline characteristics of the extended sample wherein the measurement period was set to 6 rather than 2 months.

**Number of patients**	70,304
**Gender**, n (%)	
Female	40,563 (57.7)
Male	29,724 (42.3)
Unknown	17 (0.02)
**Race**, n (%)	
White	38,308 (54.5)
Black or African American	9,509 (13.5)
Native Hawaiian or Other Pacific Islander	527 (0.75)
Asian	524 (0.75)
American Indian or Alaska Native	251 (0.36)
Other or mixed race	1,027 (1.46)
Unknown	20,158 (28.7)
**Age in years**, mean (SD)	32.0 (18.1)
**Years of education**, mean (SD)	4.69 (6.89)
**Diagnosis**, n (%)	
Major depressive disorder (MDD)	30,821 (43.8)
Bipolar disorder (BD)	17,080 (24.3)
Generalized anxiety disorder (GAD)	13,653 (19.4)
Post-traumatic disorder (PTSD)	17,572 (25.0)
Schizophrenia or schizoaffective disorder (SCZ)	6,985 (9.9)
Attention deficit hyperactivity disorder (ADHD)	18,162 (25.8)
Personality disorder (PD)	10,010 (14.2)
**Follow-up time in days**, median (IQR)	180 (120-180)
**Number of CGI-S measurements for phenotyping**, median (IQR)	11 (7-18)

Abbreviations: CGI-S, Clinical Global Impression-Severity; IQR, interquartile range; SD, standard deviation

Table S5 - Baseline characteristics of the cohort in whom PHQ-9 was measured repeatedly over a one-year phenotyping period.

**Number of patients**	4,557
**Gender**, n (%)	
Female	2467 (54.14)
Male	2089 (45.84)
Unknown	1 (0.02)
**Race**, n (%)	
White	2376 (52.14)
Black or African American	1976 (43.36)
Native Hawaiian or Other Pacific Islander	5 (0.11)
Asian	35 (0.77)
American Indian or Alaska Native	7 (0.15)
Other or mixed race	16 (0.35)
Unknown	142 (3.12)
**Age in years**, mean (SD)	31.3 (16.7)
**Diagnosis**, n (%)	
Major depressive disorder (MDD)	3197 (70.16)
Bipolar disorder (BD)	1323 (29.03)
Generalized anxiety disorder (GAD)	637 (13.98)
Post-traumatic disorder (PTSD)	868 (19.05)
Schizophrenia or schizoaffective disorder (SCZ)	621 (13.63)
Attention deficit hyperactivity disorder (ADHD)	1129 (24.78)
Personality disorder (PD)	588 (12.90)
**Follow-up time in days**, median (IQR)	180 (180-180)
**Number of PHQ-9 measurements for phenotyping**, median (IQR)	4 (4-5)

Abbreviations: IQR, interquartile range; PHQ, Patient Health Questionnaire; SD, standard deviation

Other supplementary Figure

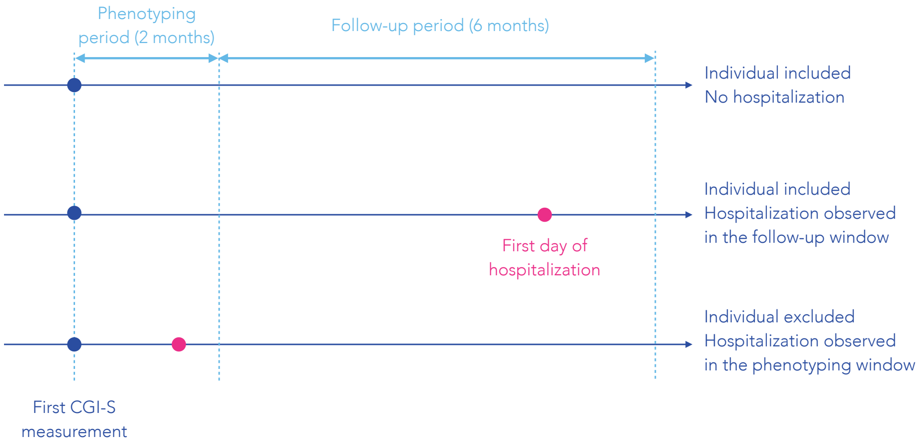

Fig. S4 – Diagram showing the timeline for different individuals and linking their first CGI-S measurements, the phenotyping and follow-up periods, and their potential first hospitalization.

References

1 Pirla S, Taquet M, Quoidbach J. Measuring affect dynamics: An empirical framework. *Behav Res Methods* 2022; published online April 5. DOI:10.3758/s13428-022-01829-0.

2 Faurholt-Jepsen M, Geddes JR, Goodwin GM, *et al.* Reporting guidelines on remotely collected electronic mood data in mood disorder (eMOOD)-recommendations. *Transl Psychiatry* 2019; **9**: 162.

3 Carr O, Saunders KEA, Tsanas A, *et al.* Variability in phase and amplitude of diurnal rhythms is related to variation of mood in bipolar and borderline personality disorder. *Sci Rep* 2018; **8**: 1649.

4 Tsanas A, Saunders KEA, Bilderbeck AC, *et al.* Daily longitudinal self-monitoring of mood variability in bipolar disorder and borderline personality disorder. *J Affect Disord* 2016; **205**: 225–33.

5 Patel R, Wee SN, Ramaswamy R, *et al.* NeuroBlu, an electronic health record (EHR) trusted research environment (TRE) to support mental healthcare analytics with real-world data. *BMJ Open* 2022; **12**: e057227.

RECORD statement
Checklist of items, extended from the STROBE statement, that should be reported in observational studies using routinely collected health data.

	Item No.	STROBE items	Location in manuscript where items are reported	RECORD items	Location in manuscript where items are reported
**Title and abstract**					
	1	(a) Indicate the study's design with a commonly used term in the title or the abstract (b) Provide in the abstract an informative and balanced summary of what was done and what was found	Abstract	RECORD 1.1: The type of data used should be specified in the title or abstract. When possible, the name of the databases used should be included.<br><br>RECORD 1.2: If applicable, the geographic region and timeframe within which the study took place should be reported in the title or abstract.<br><br>RECORD 1.3: If linkage between databases was conducted for the study, this should be clearly stated in the title or abstract.	Abstract<br><br>Abstract<br><br>N/A
**Introduction**					
Background rationale	2	Explain the scientific background and rationale for the investigation being reported	Introduction		
Objectives	3	State specific objectives, including any prespecified hypotheses	Introduction		

**Methods**					
Study Design	4	Present key elements of study design early in the paper	Methods		
Setting	5	Describe the setting, locations, and relevant dates, including periods of recruitment, exposure, follow-up, and data collection	Methods		

Participants	6	*(a) Cohort study*- Give the eligibility criteria, and the sources and methods of selection of participants. Describe methods of follow-up *Case-control study*- Give the eligibility criteria, and the sources and methods of case ascertainment and control selection. Give the rationale for the choice of cases and controls *Cross-sectional study*- Give the eligibility criteria, and the sources and methods of selection of participants<br><br>*(b) Cohort study*- For matched studies, give matching criteria and number of exposed and unexposed *Case-control study*- For matched studies, give matching criteria and the number of controls per case	Methods and supplement<br><br>N/A	RECORD 6.1: The methods of study population selection (such as codes or algorithms used to identify subjects) should be listed in detail. If this is not possible, an explanation should be provided.<br><br>RECORD 6.2: Any validation studies of the codes or algorithms used to select the population should be referenced. If validation was conducted for this study and not published elsewhere, detailed methods and results should be provided.<br><br>RECORD 6.3: If the study involved linkage of databases, consider use of a flow diagram or other graphical display to demonstrate the data linkage process, including the number of individuals with linked data at each stage.	Methods and supplement<br><br>N/A
Variables	7	Clearly define all outcomes, exposures, predictors, potential confounders, and effect modifiers. Give diagnostic criteria, if applicable.	Methods - Outcome	RECORD 7.1: A complete list of codes and algorithms used to classify exposures, outcomes, confounders, and effect modifiers should be provided. If these cannot be reported, an	Methods – Cohort definition, Exposure of interest, Outcome, Statistical Analysis

				explanation should be provided.	
Data sources/ measurement	8	For each variable of interest, give sources of data and details of methods of assessment (measurement). Describe comparability of assessment methods if there is more than one group	Methods and supplement		

Bias	9	Describe any efforts to address potential sources of bias	Methods - Statistical analysis and Secondary analyses		
Study size	10	Explain how the study size was arrived at	Methods – Data, Cohort Definition, Statistical power		
Quantitative variables	11	Explain how quantitative variables were handled in the analyses. If applicable, describe which groupings were chosen, and why	Methods		

Statistical methods	12	(a) Describe all statistical methods, including those used to control for confounding<br>(b) Describe any methods used to examine subgroups and interactions<br>(c) Explain how missing data were addressed<br>(d) *Cohort study*- If applicable, explain how loss to follow-up was addressed<br>*Case-control study*- If applicable, explain how matching of cases and controls was addressed<br>*Cross-sectional study*- If applicable, describe analytical methods taking account of sampling strategy<br>(e) Describe any sensitivity analyses	Methods – Statistical analysis and secondary analyses		
Data access and cleaning methods		..		RECORD 12.1: Authors should describe the extent to which the investigators had access to the database population used to create the study population.	Methods - Data

				RECORD 12.2: Authors should provide information on the data cleaning methods used in the study.	N/A

Linkage		..		RECORD 12.3: State whether the study included person-level, institutional-level, or other data linkage across two or more databases. The methods of linkage and methods of linkage quality evaluation should be provided.	N/A
**Results**					
Participants	13	(a) Report the numbers of individuals at each stage of the study (*e.g.*, numbers potentially eligible, examined for eligibility, confirmed eligible, included in the study, completing follow-up, and analysed)<br>(b) Give reasons for non-participation at each stage.<br>(c) Consider use of a flow diagram	Results – First paragraph	RECORD 13.1: Describe in detail the selection of the persons included in the study (*i.e.,* study population selection) including filtering based on data quality, data availability and linkage. The selection of included persons can be described in the text and/or by means of the study flow diagram.	Methods – Cohort definition and Supplement
Descriptive data	14	(a) Give characteristics of study participants (*e.g.*, demographic, clinical, social) and information on exposures and potential confounders<br>(b) Indicate the number of participants with missing data for each variable of interest (c) *Cohort study*- summarise follow-up time (*e.g.*, average and total amount)	Table 1 and Results<br><br>Methods – Statistical analysis and Results		

Outcome data	15	*Cohort study*- Report numbers of outcome events or summary measures over time<br>*Case-control study*- Report numbers in each exposure category, or summary measures of exposure<br>*Cross-sectional study*- Report numbers of outcome events or summary measures	Results and Fig. 1-3, Supplementary Table 1		
Main results	16	(a) Give unadjusted estimates and, if applicable, confounder- adjusted estimates and their precision (e.g., 95% confidence interval). Make clear which confounders were adjusted for and why they were included<br>(b) Report category boundaries when continuous variables were categorized<br>(c) If relevant, consider translating estimates of relative risk into absolute risk for a meaningful time period	Results, Fig 1-3, Supplementary Table 1, Unadjusted estimates not provided.		
Other analyses	17	Report other analyses done—e.g., analyses of subgroups and interactions, and sensitivity analyses	Results, Fig. 1-3, Supplementary Table 1		

**Discussion**					
Key results	18	Summarise key results with reference to study objectives	Discussion – First paragraph		
Limitations	19	Discuss limitations of the study, taking into account sources of potential bias or imprecision. Discuss both direction and magnitude of any potential bias	Discussion – "Limitation" paragraph	RECORD 19.1: Discuss the implications of using data that were not created or collected to answer the specific research question(s). Include discussion of misclassification bias, unmeasured confounding, missing data, and changing eligibility over time, as they pertain to the study being reported.	Discussion
Interpretation	20	Give a cautious overall interpretation of results considering objectives, limitations, multiplicity of analyses, results from similar studies, and other relevant evidence	Discussion – last paragraph		
Generalisability	21	Discuss the generalisability (external validity) of the study results	Discussion		
**Other Information**					
Funding	22	Give the source of funding and the role of the funders for the present study and, if applicable, for the original study on which the present	Acknowledgements		

		article is based			
Accessibility of protocol, raw data, and programming code		..		RECORD 22.1: Authors should provide information on how to access any supplemental information such as the study protocol, raw data, or programming code.	

*Reference: Benchimol EI, Smeeth L, Guttmann A, Harron K, Moher D, Petersen I, Sørensen HT, von Elm E, Langan SM, the RECORD Working Committee. The REporting of studies Conducted using Observational Routinely-collected health Data (RECORD) Statement. *PLoS Medicine* 2015; in press.

*Checklist is protected under Creative Commons Attribution (CC BY) license.
